# Influence of initial glucocorticoid co-medication on mortality and hospitalization in early inflammatory arthritis: an investigation by record linkage of clinical and administrative databases

**DOI:** 10.1186/s13075-022-02824-8

**Published:** 2022-06-16

**Authors:** Garifallia Sakellariou, Carlo Alberto Scirè, Federica Rumi, Greta Carrara, Anna Zanetti, Carlo Cerra, Simona Migliazza, Serena Bugatti, Carlomaurizio Montecucco

**Affiliations:** 1 Unit of Rheumatology, University of Pavia, IRCCS Policlinico San Matteo Foundation, Pavia, Italy; 2 University of Pavia, Istituti Clinici Scientifici Maugeri, Pavia, Italy; 3 Dipartimento di Scienze Mediche, Università degli studi di Ferrara, Clinica di Reumatologia, Ferrara, Italy; 4 Epidemiology Unit, Italian Society for Rheumatology (SIR), Milan, Italy; 5 Department of Medical Sciences, Section of Hematology and Rheumatology, University of Ferrara and Azienda Ospedaliero-Universitaria S. Anna, via A. Moro 8, 44124 Cona, FE Italy; 6 Information System and Management Control, Local Health Authority (ATS), Pavia, Italy

**Keywords:** Rheumatoid arthritis, Early arthritis, Glucocorticoids, Mortality, Administrative Healthcare Databases

## Abstract

**Background:**

While low-dose oral glucocorticoids (GCs) are recommended in the management of early arthritis, their impact on mortality is unclear. The aim of this study is to evaluate the effect of GCs on mortality in patients with early arthritis, by linking clinical and administrative databases.

**Methods:**

The study included patients with new-onset rheumatoid arthritis (RA) or undifferentiated arthritis (2005–2010), who received DMARDs (MTX in RA or UA with poor prognosis, hydroxychloroquine in UA) and were alive at the second year of follow-up. Low-dose GCs could be prescribed. Clinical and administrative data were linked from Administrative Health Databases (AHD) of the corresponding province, which provided us with information on drug delivery, comorbidities, hospitalization, and mortality. The effect of GCs in the first year was defined using a dichotomous variable or a 3-level categorization (not delivered, ≤7.5 mg/day, or >7.5 mg/day of prednisone) on all-cause mortality, assessed with Cox regression, either crude or adjusted for age, gender, Charlson Comorbidity Index (CCI) or single comorbidities, ACPA, HAQ, and MTX in the first year. A secondary analysis of the effect of GCs on related hospitalizations (for cardiovascular events, diabetes, serious infections, osteoporotic fractures) was also carried.

**Results:**

Four hundred forty-nine patients were enrolled (mean age 58.59, RA 65.03%) of which 51 (11.36%) died during the study. The median (IQR) follow-up was equal to 103.91 (88.03–126.71) months. Treatments with GCs were formally prescribed to 198 patients (44.10%) at ≤7.5 mg/day, although by the end of the study such treatments were received by 257 patients (57.24%); 88 patients (19.6%) were treated with GCs at >7.5 mg/day. In adjusted analyses, the GC delivery (HR, 95% CI 1.35 (0.74, 2.47)) did not significantly predict mortality — both at a low (HR, 95% CI 1.41 (0.73, 2.71)) and at a high (HR, 95% CI 1.23 (0.52, 2.92)) dosage. When “all-cause hospitalization” was used as an outcome, the analysis did not show a difference between patients receiving GC and patients not receiving GC.

**Conclusion:**

In patients with early inflammatory arthritis, the initial GC dose was higher than that prescribed by rheumatologists; however, on background treatment with DMARDs, GC treatments did not seem to increase mortality and hospitalizations.

**Supplementary Information:**

The online version contains supplementary material available at 10.1186/s13075-022-02824-8.

## Background

Rheumatoid arthritis (RA) is a chronic inflammatory disease primarily involving synovial joints, in which persistent inflammation leads to joint destruction and irreversible disability. Despite a prevalent articular involvement, RA is a systemic condition in which chronic inflammation drives the development of comorbidities. As a consequence, patients with RA experience increased mortality compared to the general population [1]. This excess of mortality has been particularly related with an increase in death for cardiovascular events, infections, and neoplasms [2, 3].

In the last two decades, the optimization of the management of RA and inflammatory arthritis with early diagnosis, early intensive treatment, and new therapeutic agents has driven a major improvement in medium-term disease-related outcomes, such as functional disability and radiographic progression [4]. Treatment strategies have also shown to decrease the risk of premature death. Indeed, treatments with methotrexate (MTX) and other disease-modifying anti-rheumatic drugs (DMARDs) decrease all-cause and cardiovascular mortality [5, 6]. In line with this finding, an early clinical response also seems to play a positive effect over mortality, with patients achieving early remission showing a lower mortality, when compared to those having an active disease [7, 8].

While the beneficial role of DMARDs on hard outcomes has been demonstrated, several studies have consistently shown a detrimental dose-dependent effect of glucocorticoids (GCs) on premature death [9–11]. This is in contrast with the rapid effect of GCs, which lead to the fast achievement of low disease activity and a deeper suppression of synovitis. Such rapid disease control may account for the long-lasting beneficial effects on radiographic damage [12]. Moreover, GC co-medication has shown to abolish the positive effect of DMARDs on mortality [13]. Besides their impact on mortality, GCs manifest their detrimental effects also on other outcomes, including GC-related hospitalizations [14]. In contrast with these reports, however, very recent data from the COBRA study, whose treatment strategy included an initial high dose of GCs randomly allocated to patients, demonstrated no increased risk of death compared to the general population after 23 years, supporting the benefits of an early intensive treatment regimen with GC [15].

When the impact of GCs on mortality and hospitalization is examined, confounding cannot be excluded if high doses of GCs are prescribed to patients who are affected by severe diseases and, therefore, have a high risk of death. Besides, the increased mortality apparently promoted by GC emerges from observational studies in which adherence to the proposed treatment regimen could not be verified. Based on the controversial evidence on their effects, although GCs are recommended in the treatment of early arthritis, they are typically advised at a low dosage for short periods [16].

Recent studies addressed the question of whether the improvement of disease outcomes in RA might have resulted in a positive impact over the excess of mortality, but results have been inconsistent. Additionally, a meta-analysis demonstrated that the gap in mortality between subjects with RA and healthy individuals has yet to be filled [17], while other studies have shown no increase in mortality in more recent populations [18, 19]. Moreover, while the attention on early referral has driven the spread of dedicated early arthritis clinics, the evidence supporting the superiority of this organizational effort, in particular on hard outcomes, compared to standard management, is still limited [20]. One of the main difficulties in studying the mortality in a standard observational context is the fact that the assessment of long-term outcomes requires following patients for a long time, and often patients are lost during the follow-up and there is a lack of information on the causes of their death, which strongly affects the statistical power in the subsequent data analysis.

On the other hand, AHD — instituted to collect data for administrative and management purposes — provide a large amount of information on the use of sanitary resources, including pharmacological prescriptions, information on vital status, and the causes of death. AHD can be used to perform epidemiological research, although their main limitation is the lack of clinically relevant information [21, 22]. A possible way to overcome these constraints could be the union between AHD and databases constructed for clinical research [23].

This study aims to evaluate the impact of initial GC co-medication on background treatment with DMARDs on all-cause mortality, through record linkage between AHD and the clinical database from an early arthritis clinic. The target population are patients affected by early inflammatory arthritis and treated with a tight control strategy, which aims at low disease activity. Besides, the study also tries to explore the impact of GCs on hospitalizations which could be related to these treatments.

## Methods

### Clinical cohort

Consecutive patients with RA or undifferentiated arthritis (UA) were enrolled at the Early Arthritis Clinic (EAC) of the IRCCS Policlinico San Matteo Foundation, Pavia, Italy [24]. The EAC was instituted in 2005 to include consecutive patients, referred by general practitioners, with new-onset inflammatory arthritis (symptom duration <12 months) from the Pavia district. Referral criteria included (1) joint stiffness of 30 min or more, (2) presence of swollen joints, and (3) positive squeezing test at the metacarpophalangeal or metatarsophalangeal joints [25]. At the first evaluation, rheumatoid factor (RF), anti-citrullinated protein antibodies (ACPA), erythrosedimentation rate (ESR), and C-reactive protein (CRP) levels were dosed. Patients underwent a joint count on 66/68 joints and the main clinimetric measures were recorded, including visual analogue scale (VAS) for pain, general health, and patient and physician global assessment; patients also carried out the Italian version of the Health Assessment Questionnaire (HAQ) [26]. The interval from the first symptom to diagnosis was recorded.

The EAC population is composed by a historical cohort (2005–2010) and a more recent cohort, different in terms of classification and therapeutic protocol. All patients seen after 2010 received a low dose of GCs and a different DMARD dosage. There were several substantial differences between the two populations that would have made the two cohorts not comparable. For this reason, to answer our research question on a homogeneous population, we included only the historical cohort, in which RA was classified based on the 1987 ACR criteria [27]. Patients affected by RA or UA with poor prognostic factors received MTX from the dose of 10 mg/week [28]. Patients with UA received hydroxychloroquine (HCQ) (400 mg/day for 2 months, 200 mg/day afterwards). Patients were seen every 2 months in the first semester and every 3 afterwards; the treatment was adjusted to reach a disease activity score (DAS) <2.4. In case of active disease at subsequent evaluations, HCQ was substituted with MTX in patients with UA, while the dose could be escalated up to 20 mg/week or the maximum tolerated in patients already receiving MTX. When HCQ or MTX was contraindicated, alternative DMARDs were prescribed, for instance, leflunomide, sulfasalazine, or cyclosporine. In the case of persistently active disease and if indicated, patients received biologic (b) DMARDs. Based on the clinical decision and in the context of an open-label trial for part of the patients, a low dose (12.5 mg/day for 2 weeks, then 6.25 mg/day for 2 years) of oral prednisone was prescribed [29].

The present study was conducted according to the Declaration of Helsinki and approved by the local Ethics Committee (approval number P-20130002166); all patients provided their written informed consent before the beginning of the study.

### Administrative database

The AHD of the province of Pavia is an automated system of databases, instituted in 2004, consisting of (1) an archive of all residents in Pavia district with access to the National Health Service (NHS), reporting demographic and administrative data, including information on vital status and causes of death; (2) all the exemptions for chronic disease; (3) hospital discharge forms reporting diagnoses related to hospitalization; and (4) all outpatient drug prescription reimbursable by the NHS. Diagnoses are coded according to the International Classification of the Disease (ICD) 9, while drugs by the Anatomic Therapeutic Chemical classification (ATC). The ICD and ATC codes adopted for this analysis are reported in tables S1 and S2.

Data from the clinical and administrative databases were linked using a unique identifier (tax code), which provided a deterministic record linkage between them [11]. For this study, information on drugs, comorbidities, hospitalizations, and mortality was obtained from the AHD. Indeed, in the Italian National Health System, the prescriber is the general practitioner. Each drug prescription is recorded in the AHD once it is delivered to a pharmacy and the patient obtains the drug. For this reason, the AHD can be seen as a reliable source, reflecting more reliably the drug intake. The prescriptions of GC in the clinical database and in the AHD were compared. The mean daily dose of GC in prednisone equivalents was calculated as mean mg per day of prednisone equivalents. In the analyses, the use of GC in the first year of observation was analyzed both as a dichotomous variable and by creating 3 categories: (1) no GC (reference), (2) mean daily dose in the first year of observation ≤7.5 mg, and (3) mean daily dose in the first year of observation >7.5 mg [30]. Moreover, the adherence to GC in the first year of treatment in patients receiving at least one prescription was calculated as the proportion of days covered, given a defined daily dose (DDD) of 6.25 mg/day of prednisone.

The AHD was used to build the Charlson Comorbidity Index (CCI), dichotomized into rheumatologic (reference) and non-rheumatologic comorbidities [31]. Moreover, since the comorbidities defined in the CCI might not reliably reflect those influencing the choice to administer GC, we not only assessed the prevalence of single comorbidities with CCI but also evaluated the prevalence of single comorbidities such as hypertension, severe dyslipidemia, serious infections, and severe osteoporosis.

### Statistical analysis

Descriptive statistics reporting frequencies, mean with standard deviation (sd), median, and interquartile range (IQR) were performed as appropriate.

The outcome of interest was all-cause mortality occurring at any time during observation.

Patients were followed from the first evaluation at the EAC until death or until 31/12/2016. The analysis was limited to patients who were alive after 2 years of follow-up and received at least a DMARD prescription within the first year of observation; in this manner, we could exclude all those patients who had severe comorbidities leading to premature death and accounting for not prescribing GC. However, to exclude survival bias, we performed an additional analysis including also patients dying within the second year of observation. By applying separate models including a dichotomous and the 3-level categorization, the predictive value of GC given in the first year after diagnosis over mortality was tested through Cox regression models, both crude and adjusted for gender, age, HAQ, CCI (dichotomous), use of MTX in the first year from diagnosis, and ACPA (dichotomous). Additional models, in which CCI was substituted by single more prevalent comorbidities (diabetes, myocardial infarction, neoplasms, arterial hypertension), were also tested. Furthermore, the interaction between CCI and GC was tested to take into account the potential impact of comorbidities on the prescription of GC. To exclude a different effect of GC after the first year, the primary analysis was repeated using the mean dosage for all the observation period.

Another analysis was performed using as outcome the hospitalizations occurring after the first year of observation and potentially related to GC (i.e., myocardial infarction, cerebrovascular accidents, serious infections, vertebral and femoral fractures), applying separate models including the dichotomous and the 3-level categorization of GC therapy. The ICD codes used to define GC-related hospitalizations are presented in supplementary table S3.

## Results

### Descriptive analyses

The present analysis included a total of 449 patients with RA or UA, who were enrolled before October 1, 2010, had a DMARD prescription in the first year, were alive until the end of the second year of observation, and possessed complete data in the clinical database. Three hundred twenty-six (72.61%) were female, with a mean (sd) age of 58.59 (14.16) years; 292 (65.03%) fulfilled the 1987 criteria for RA. However, when sufficient data to apply the 2010 criteria were available, 55/127 (43.3%) additional UA subjects could be classified as RA. The median (interquartile range, IQR) follow-up was 103.91 (88.03–126.71) months; however, 51 patients (11.36%) died during the observation. Complete demographic and clinical characteristics of the population are reported in Table 1.

**Table 1:** Demographic and clinical features of the included population

N		449
RA/UA (n, %)		292/157 (65.03/34.97)
M/F (n, %)		123/326 (27.39/72.61)
Age, years (mean, sd)		58.59 (14.16)
RF or ACPA (n, %)		154 (37.65)
HAQ (median, IQR)		1 (0.625−1.625)
SJC 28 (median, IQR)		6 (3−10)
TJC 28 (median, IQR)		6 (2−11)
DAS28 (mean, sd)		4.75 (1.29)
ESR (median, IQR)		22 (13−39.75)
CRP (median, IQR)		0.69 (0.31−2.1)
VAS PGA (median, IQR)		57.5 (40.25−76.75)
VAS pain (median, IQR)		54 (40−80)
VAS GH (median, IQR)		56 (50−75)
VAS EGA (median, IQR)		40 (27−50.75)
Charlson Comorbidity Index (median, IQR)		1 (1−1)
Arterial hypertension (n, %)		117 (26.06)
Diabetes (n, %)		40 (8.91)
Neoplasm (n, %)		23 (5.12)
Myocardial infarction (n, %)		12 (2.67)
GC (n, %)		257 (57.24)
GC ≤7.5 mg/day (n, %)		169 (37.64)
GC >7.5 mg/day (n, %)		88 (19.6)
GC daily dose (median, IQR)	Overall sample	2.31 (0−6.87)
	Patients receiving glucocorticoids	6.45 (4.17−7.83)
MTX (n, %)^a^		312 (69.49)
bDMARDs (n, %)^a^		10 (2.23)
Deaths (n, %)		51 (11.36)
Follow-up, months (median, IQR)		103.91 (88.03−126.71)

^a^Proportions refer to the first year of observation. *RA* rheumatoid arthritis, *UA* undifferentiated arthritis, *sd* standard deviation, *IQR* interquartile range, *RF* rheumatoid factor, *ACPA* anti-cyclic citrullinated peptide antibodies, *HAQ* Health Assessment Questionnaire, *TJC 28* tender joint count on 28 joints, SJC 28 swollen joint count on 28 joints, *DAS28* disease activity score on 28 joints, *VAS* visual analogue scale, *PGA* patient’s global assessment, *EGA* evaluator global assessment, *GC* glucocorticoid, *MTX* methotrexate, *bDMARDs* biologic disease-modifying antirheumatic drugs

The most prevalent comorbidity was arterial hypertension; severe dyslipidemia, infections, and severe osteoporosis were not included in subsequent models, as they affected less than 10 patients each. GCs were given in the AHD to 257 (57.24%) patients, 169 (37.65%) at dosage ≤ 7.5 mg/day, while 88 (19.6%) >7.5 mg/day. However, analyzing data from the clinical database, GCs had been prescribed by the rheumatologist only to 198 (44.10%) patients. When comparing the prescription of GCs in the clinical database and AHD, we observed that, among the patients to whom GCs were prescribed, 188/198 patients (94.95%) had at least one prescription in the AHD; on the contrary, among the patients to whom GCs were not indicated, 62/240 patients (25.83%) had at least one prescription in the AHD. In those taking GC despite not being prescribed, the median (IQR) daily dose was 4.43 (1.98–7.00). Based on the data from the ADH, the adherence to GC was also calculated in the subgroup of patients receiving GC; the number of subjects with an adherence below 80% was 117/257 (45.52%).

### Primary outcome

**Fig. 1:**
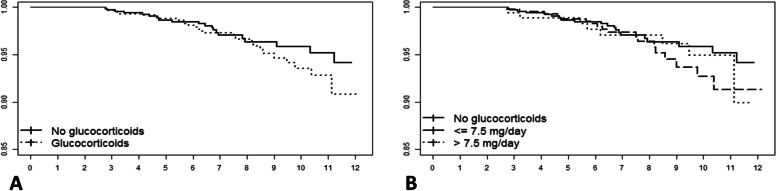
Cox regression models. **A** Glucocorticoid treatment defined as a categorical variable. Adjustment for age, gender, anti-cyclic citrullinated peptide antibodies, Health Assessment Questionnaire, Charlson Comorbidity Index, and methotrexate in the first year. **B** Three-level definition of glucocorticoid treatment. Adjustment for age, gender, anti-cyclic citrullinated peptide antibodies, Health Assessment Questionnaire, Charlson Comorbidity Index, and methotrexate in the first year

In both crude and adjusted analyses, the prescription of GCs did not associate with increased mortality. In the same model, age and the CCI were significant predictors, while administration of MTX in the first year, compared to other DMARDs, resulted in a reduced risk (Table 2, Fig. 1). The same analysis including patients dying in the first 2 years provided similar results (Supplementary Table S4).

**Table 2:** Cox regression analysis on mortality, 2-level definition of corticosteroid treatment, including CCI (non-rheumatologic comorbidities vs rheumatologic comorbidities only)

	HR (95% CI)	Adj HR (95% IC)
No GC	Ref	Ref
GC	0.99 (0.57, 1.72)	1.35 (0.74, 2.47)
Female gender		0.65 (0.36, 1.18)
Age	***-***	***1.11 (1.07, 1.15)***
HAQ	-	1.36 (0.90, 2.05)
CCI >1	***-***	***2.29 (1.23, 4.26)***
MTX	-	***0.43 (0.21, 0.89)***
ACPA	-	1.54 (0.71, 3.34)

*GC* glucocorticoid, *HR* hazard ratio, *CI* confidence interval, *HAQ* Health Assessment Questionnaire, *CCI* Charlson Comorbidity Index, *MTX* methotrexate, *ACPA* anti-cyclic citrullinated S

When evaluating GCs based on the dose, we can see that both doses in crude analyses did not predict mortality as adjusted analyses yielded to the same results. In the same model, age and the CCI were also significant predictors, while MTX had a protective effect (Table 3, Fig. 1).

**Table 3:** Cox regression analysis on mortality, 3-level definition of corticosteroid treatment, including CCI (non-rheumatologic comorbidities vs rheumatologic comorbidities only)

	HR (95% CI)	Adj HR (95% IC)
No GC	Ref	Ref
GC ≤7.5 mg/day	0.99 (0.53, 1.83)	1.41 (0.73, 2.71)
GC >7.5 mg/day	0.99 (0.47, 2.10)	1.23 (0.52, 2.92)
Female gender	-	0.65 (0.36, 1.18)
Age	*-*	***1.11 (1.07, 1.15)***
HAQ	-	1.35 (0.90, 2.04)
CCI >1	*-*	***2.29 (1.23, 4.27)***
MTX	-	***0.44 (0.21, 0.93)***
ACPA	-	1.58 (0.72, 3.46)

*GC* glucocorticoid, *HR* hazard ratio, *CI* confidence interval, *HAQ* Health Assessment Questionnaire, *CCI* Charlson Comorbidity Index, *MTX* methotrexate, *ACPA* anti-cyclic citrullinated peptide antibodies

The model including the mean GC dose throughout the follow-up showed similar results, in particular, there was no significant increase in mortality due to GC (Supplementary table S5).

Furthermore, in the analyses in which CCI was substituted by single comorbidities, GC treatment did not result in a significant prediction of mortality. When separating GC depending on dosage, both low doses and higher doses did not result in increased mortality. In both models, age, diabetes, and myocardial infarction were significant predictors (Tables 4 and 5, Fig. 1). The evaluation of the interaction between CCI and GC prescription was not included in the models because it resulted in no statistically significant effect.

**Table 4:** Cox regression analysis on mortality, 2-level definition of corticosteroid treatment, including single comorbidities

	Adj HR (95% IC)
No GC	Ref
GC	1.84 (0.94, 3.59)
Female gender	0.73 (0.40, 1.35)
Age	***1.10 (1.06, 1.14)***
HAQ	1.09 (0.70, 1.71)
Arterial hypertension	1.13 (0.60, 2.10)
Diabetes	***4.59 (2.15, 9.82)***
Neoplasm	0.40 (0.05, 2.99)
Myocardial infarction	***3.08 (1.05, 9.04)***
MTX	0.51 (0.25, 1.07)
ACPA	1.39 (0.64, 3.02)

*GC* glucocorticoid, *HR* hazard ratio, *CI* confidence interval, *HAQ* Health Assessment Questionnaire, *MTX* methotrexate, *ACPA* anti-cyclic citrullinated peptide antibodies

**Table 5:** Cox regression analysis on mortality, 3-level definition of corticosteroid treatment, including single comorbidities

	Adj HR (95% IC)
No GC	Ref
GC ≤ 7.5 mg/day	1.84 (0.90, 3.75)
GC > 7.5 mg/day	1.85 (0.74, 4.65)
Female gender	0.73 (0.40, 1.35)
Age	***1.10 (1.06, 1.14)***
HAQ	1.09 (0.70, 1.71)
Arterial hypertension	1.13 (0.60, 2.10)
Diabetes	***4.59 (2.14, 9.84)***
Neoplasm	0.40 (0.05, 2.99)
Myocardial infarction	***3.09 (1.04, 9.14)***
MTX	0.51 (0.24, 1.09)
ACPA	1.38 (0.63, 3.05)

*GC* glucocorticoid, *HR* hazard ratio, *CI* confidence interval, *HAQ* Health Assessment Questionnaire, *MTX* methotrexate, *ACPA* anti-cyclic citrullinated peptide antibodies

### Secondary outcome

One hundred thirteen out of 449 patients experienced at least a GC-related hospitalization during the follow-up; among these patients, 65/257 (24.9%) received GC in the first year of observation, while 49/192 (25.5%) did not. The two proportions were not significantly different (*p* = 0.969). The most common cause of hospitalization was cardiovascular events (84 patients), followed by diabetes (38 patients). In both crude and adjusted analyses, the prescription of GC did not associate with increased hospitalization for all causes. In the same model, age, gender, HAQ, and the CCI were significant predictors (Table 6). The same analysis including patients dying in the first 2 years led to similar results (Supplementary table S6).

**Table 6:** Cox regression analysis on GC-related hospitalization, 2-level definition of corticosteroid treatment, including CCI (non-rheumatologic comorbidities vs rheumatologic comorbidities only)

	HR (95% CI)	Adj HR (95% IC)
No GC	Ref	Ref
GC	0.95 (0.65, 1.37)	1.08 (0.72, 1.61)
Female gender	-	***0.41 (0.27, 0.60)***
Age	***-***	***1.07 (1.05, 1.09)***
HAQ	-	***1.34 (1.02, 1.76)***
CCI >1	***-***	***1.76 (1.14, 2.71)***
MTX	-	1.06 (0.63, 1.80)
ACPA	-	1.58 (0.95, 2.61)

*GC* glucocorticoid, *HR* hazard ratio, *CI* confidence interval, *HAQ* Health Assessment Questionnaire, *CCI* Charlson Comorbidity Index, *MTX* methotrexate, *ACPA* anti-cyclic citrullinated peptide antibodies

When evaluating GC based on the dose, both doses in crude analyses did not predict hospitalization. Adjusted analyses yielded to the same result. In the same model, age, gender, HAQ, the CCI, and ACPA positivity were significant predictors (Table 7).

**Table 7:** Cox regression analysis on GC-related hospitalization, 3-level definition of corticosteroid treatment, including CCI (non-rheumatologic comorbidities vs rheumatologic comorbidities only)

	HR (95% CI)	Adj HR (95% IC)
No GC	Ref	Ref
GC ≤ 7.5 mg/day	1.06 (0.71, 1.59)	1.30 (0.84, 1.99)
GC > 7.5 mg/day	0.73 (0.42, 1.27)	0.71 (0.38, 1.31)
Female gender	-	***0.41 (0.28, 0.61)***
Age	*-*	***1.07 (1.05, 1.09)***
HAQ	-	***1.31 (1, 1.72)***
CCI >1	*-*	***1.80 (1.17, 2.78)***
MTX	-	1.15 (0.68, 1.95)
ACPA	-	***1.81 (1.07, 3.05)***

*GC* glucocorticoid, *HR* hazard ratio, *CI* confidence interval, *HAQ* Health Assessment Questionnaire, *CCI* Charlson Comorbidity Index, *MTX* methotrexate, *ACPA* anti-cyclic citrullinated peptide antibodies

## Discussion

The evaluation of long-term outcomes in observational studies of inflammatory arthritis is often limited by attrition, missing information, and the need for a long-term follow-up. Due to these constraints, many studies on the impact of treatment on mortality derive from historical cohorts, which is also quite common for GCs [3, 5, 11]. In our study, we examined incident cases of early inflammatory arthritis, treated in line with more recent treatment approaches. Our sample was composed by both RA and UA, and the features of this population likely reflect a more modern concept of RA. Attrition and missing data were overcome through the record linkage with AHD, while the scarce detail of clinical information in the AHD was compensated using the clinical database. Based on a median (IQR) observation of 8.65 (7.33–10.55) years, only 11% of patients died with lower mortality compared to previous reports; the inclusion of patients with UA might have influenced this result though [13].

In the context where the study was performed, the rheumatologist provides advice for treatment, but the final prescriber is the general practitioner. Descriptive analyses showed a relevant discrepancy between the rheumatologist’s prescription and the real GC intake. Despite being prescribed only low doses, almost one-fifth of our sample took medium-high dosages, and one out of four patients not meant to receive GC had at least one prescription. Although it was not possible to analyze all the potential indications of GCs, the median daily intake suggests a continuous rather than on-demand administration. In our population, only 2 patients had a coded diagnosis of chronic obstructive pulmonary disease in the AHD; thus, this comorbidity does not seem to justify GC prescription. When treatment is defined based on the secure record of the AHD, there is a significant gap between what the rheumatologist thinks to have prescribed and what happens in reality, with a tendency towards higher intake. When prescribing GCs, the main concern is typically non-adherence [32]; however, the behavior of subjects not meant to receive GC is currently not known [33, 34]. This aspect should be taken into consideration when comparing the results of the present analysis with previous studies, which are only based on clinical databases.

In our cohort, initial GC co-medication in a DMARD treat-to-target regimen did not significantly raise the risk of death, as it did not increase, and the rate of GC-related hospitalizations. Previous reports demonstrated greater mortality and the moderation of the protective effect of DMARDs by GC [12, 35]. In existing studies, the effect of GCs on the increased risk seems to act with a dose-dependent trend; the minimum daily dose associated with increased mortality was 8–15 mg/day, and the negative result in patients receiving low-dose in our cohort is in line with this finding [9]. Although analyses on patients on higher dosages may not be generalizable due to the small sample size, a different stage of the disease and a treatment strategy, which implies subsequent GC suspension, might contribute to this difference.

While several studies demonstrated lower mortality compared to the past in patients with early arthritis, the administration of GCs still increased mortality also in these populations [35]. Once again, the length of the GC medication might be relevant. Very recently, the 23-year follow-up of the COBRA study has shown no increased risk compared to the general population, suggesting an overall beneficial role of the initial intensive treatment with GC, although the effect of these drugs was not specifically addressed [14].

Our results on the rate of hospitalization for possible complications of the GC treatment are also discordant with previous studies, which report a higher risk of hospitalization for adverse events [14, 36, 37]. Despite the impossibility of determining the different causes, this difference might be interpreted as the consequence of a shorter course of GCs at disease onset in our population, and eventually by the use of a composite definition of hospitalization.

When analyzing the effect of GCs on mortality and hospitalization in RA, it is extremely hard to fully eliminate the confounding in accordance with which more severe and comorbid patients receive more frequently GCs. We tried to address these issues at our best, excluding patients dying prematurely and those not receiving DMARDs, while CCI, comorbidities, and the HAQ were introduced as covariates in the analysis to measure the disease activity and severity. In the analyses on mortality, a possible interaction between the prescription of GCs and comorbidities was also tested, resulting in no significant effect. While the discrepancy between the results in our population and historical cohorts can be justified by earlier diagnosis, historical trends, and different treatment approaches, the difficulties in interpreting selection bias could also account for discrepant results.

All our population received csDMARDs; however, in line with previous studies, MTX was related to a lower risk of death in this context [5]. This indirectly confirms the inclusion of a sample representing the features of the severity of RA patients and supports the generalizability of our findings.

The main limitation of the study is represented by the limited number of events during the follow-up. This might not have allowed a precise estimation of the effect of predictors on the outcomes and did not allow the analysis of mortality by cause. As a consequence, minor increases of mortality or hospitalization due to GCs cannot be excluded. Although all the main confounders have been taken into account, we cannot fully exclude other factors introducing bias.

## Conclusions

Recommendations on the treatment of RA support the use of GCs, although a short-term treatment at a low dose is advised, particularly in light of adverse effects and increased mortality [30]. This evidence derives mainly from historical populations, while data on contemporary cohorts are still scarce. The results of our study increase the evidence supporting the use of GCs according to the pattern proposed by current recommendations.

## Supplementary Information

**Additional file 1: Table S1. **ATC codes used to identify treatments in the AHD**. Table S2. **Codification of comorbidities for the construction of the Charlson comorbidity index and the prevalence of single comorbidities. **Table S3. **Codification of causes of hospitalization, potentially related to GC treatment. **Table S4. **Cox regression analysis on mortality including patients dying in the first two years, 2-level definition of corticosteroid treatment, including CCI (non-rheumatologic comorbidities vs rheumatologic comorbidities only). **Table S5. **Cox regression analysis on mortality, GC included as mean daily dose throughout the follow-up, including CCI (non-rheumatologic comorbidities vs rheumatologic comorbidities only). **Table S6. **Cox regression analysis on GC-related hospitalization including patients dying in the first two years, 2-level definition of corticosteroid treatment, including CCI (non-rheumatologic comorbidities vs rheumatologic comorbidities only).

## Data Availability

The datasets used and/or analyzed during the current study are available from the corresponding author on reasonable request.

## Abbreviations

RA: Rheumatoid arthritis
DMARDs: Disease-modifying antirheumatic drugs
MTX: Methotrexate
GC: Glucocorticoid
AHD: Administrative Healthcare Database
UA: Undifferentiated arthritis
EAC: Early arthritis clinic
ACPA: Anti-cyclic citrullinated peptide antibodies
ESR: Erythrosedimentation rate
CRP: C-reactive protein
VAS: Visual analogue scale
HAQ: Health Assessment Questionnaire
HCQ: Hydroxychloroquine
NHS: National Health System
ICD: International Classification of the Diseases
ATC: Anatomic Therapeutic Chemical classification
DDD: Defined daily dose
CCI: Charlson Comorbidity Index
SD: Standard deviation
IQR: Interquartile range
HR: Hazard ratio
CI: Confidence interval
