# Supplementary material for: Influence of initial glucocorticoid co-medication on mortality and hospitalization in early inflammatory arthritis: an investigation by record linkage of clinical and administrative databases
Source: Arthritis Res Ther. 2022 Jun 16;24:144. doi: 10.1186/s13075-022-02824-8 (PMC9204953; doi:10.1186/s13075-022-02824-8)
Supplement: Supplementary file 1 — Additional file 1: Table S1. ATC codes used to identify treatments in the AHD. Table S2. Codification of comorbidities for the construction of the Charlson comorbidity index and the prevalence of single comorbidities. Table S3. Codification of causes of hospitalization, potentially related to GC treatment. Table S4. Cox regression analysis on mortality including patients dying in the first two years, 2-level definition of corticosteroid treatment, including CCI (non-rheumatologic comorbidities vs rheumatologic comorbidities only). Table S5. Cox regression analysis on mortality, GC included as mean daily dose throughout the follow-up, including CCI (non-rheumatologic comorbidities vs rheumatologic comorbidities only). Table S6. Cox regression analysis on GC-related hospitalization including patients dying in the first two years, 2-level definition of corticosteroid treatment, including CCI (non-rheumatologic comorbidities vs rheumatologic comorbidities only). [file 13075_2022_2824_MOESM1_ESM.docx]

**Influence of initial glucocorticoid co-medication on mortality and hospitalization in early inflammatory arthritis. An investigation by record linkage of clinical and administrative databases – Supplementary material**

**Table S1. ATC codes used to identify treatments in the AHD.**

| **Drug** |  | **ATC code** |
| --- | --- | --- |
| csDMARDs | Methotrexate | L01BA01 |
|  | Hydroxychloroquine | P01BA02 |
|  | Sulphasalazine | A07EC01 |
|  | Leflunomide | L04AA13 |
|  | Cyclosporine | L04AD01 |
| bDMARDs | Infliximab | L04AB02 |
|  | Etanercept | L04AB01 |
|  | Adalimumab | L04AB04 |
|  | Golimumab | L04AB06 |
|  | Certolizumab | L04AB05 |
|  | Abatacept | L04AA24 |
|  | Tocilizumab | L04AC07 |
|  | Rituximab | L01XC02 |
| Glucocorticoids | Prednisone | H02AB07 |
|  | Methylprednisolone | H02AB04 |
|  | Deflazacort | H02AB13 |

CsDMARDs: conventional synthetic disease modifying antirheumatic drugs; bDMARDs: biological disease modifying antirheumatic drugs.

**Table S2. Codification of comorbidities for the construction of the Charlson comorbidity index and the prevalence of single comorbidities.**

| ***Charlson Comorbidity Index*** | | ***ICD 9*** | | ***Exemption code*** |
| --- | --- | --- | --- | --- |
| Myocardial infarction | | | 410, 412 | 002.414 |
| Congestive Heart Failure | | | 428 | 021.428 |
| Peripheral vascular disease | | | 441, 4439, 7854, V434, 3848 | 002.441.2, 002.441.7, 002.441.9,002.442, 002.444, 002.447.1 |
| Cerebrovascular disease | | | 430, 431, 432, 433, 434, 435, 436, 437, 438 | 002.433, 002.434, 002.437 |
| Dementia | | | 290 | 011.290.0, 011.290.1, 011.290.2, 011.290.4, 011.291.1, 011.294.0, 029.331.0 |
| Chronic pulmonary disease | | | 490, 491, 492, 493, 494, 495, 496, 500, 501, 502, 503, 504, 505, 5064 | 024.518.81 |
| Peptic ulcer disease | | | 531, 532, 533, 534 | - |
| Mild liver disease | | | 5712, 5714, 5715, 5716 | 008.571.2, 008.571.5, 008.571.6, 016 |
| Diabetes | | | 2500, 2501, 2502, 2503, 2507 | 013.250 |
| Diabetes with chronic complications | | | 2504, 2505, 2506 | - |
| Hemiplegia or paraplegia | | | 3441, 3420, 3421, 3422, 3423, 3424, 3425, 3426, 3427, 3428, 3429 | - |
| Renal Disease | | | 582, 585, 586, 588, 5830, 5831, 5832, 5833, 5834, 5835, 5836, 5837 | 031.403, 031.404, D31.403, D31.404, 023 |
| Malignancy | | | 14, 15, 16, 18, 170, 171, 172, 174, 175, 176, 177, 178, 179, 190, 191, 192, 193, 194, 195 | 048 |
| Leukemia | | | 2024, 2031, 204, 205, 206, 207, 208 | - |
| Lymphoma | | | 200, 201, 2020, 2021, 2022, 2023, 2025, 2026, 2027, 2028, 2029, 2030, 2038 | - |
| Moderate or severe liver disease | | | 5722, 5723, 5724, 5725, 5726, 5727, 5728, 4560, 4561, 4562 | - |
| Metastatic solid tumor | | | 196, 197, 198, 199 | - |
| AIDS | | | 042, 043, 044 | 020 |
| ***Other comorbidities*** |  | | | |
| Arterial hypertension | | | - | 031 |
| Severe dyslipidemia | | | - | 025 |

**Table S3. Codification of causes of hospitalization, potentially related to GC treatment.**

| ***Cause of Hospitalization*** | ***ICD 9*** | |
| --- | --- | --- |
| Myocardial infarction, acute cerebrovascular events, other acute cardiovascular events | | 410*, 411*, 413* and 121-123, 140  431*, 433-435* and 014,015  415.1, 415.11, 415.19, 426.0, 426.12-426.13, 426.51-426.52, 426.54, 427.1, 427.4, 427.41-427.42, 427.5,  428*, 430*-436*, 441.0*, 441.1, 441.3, 453.0, 453.2-453.3, 453.8, 785.51 and 078, 087, 106, 110-114, 127-  128, 138-139, 515, 518, 524-525, 528, 535-536, 547-559 |
| Decompensated diabetes | | 249*, 250*, 357.2, 362.01–06, 366.41 |
| Severe infections | | 049* and 320* meningitis  054.3 and 323* encephalitis  681*-682* cellulitis  421* endocarditis  481*-482* pneumonia  590* pyelonephritis  711* septic arthritis  730.0*-730.2* osteomyelitis  038* and 790.7 bacteraemia |
| Vertebral fracture | | 806.8, 805.00-4, 805.8, 805, 8050, 8052, 8054,8058  Procedures 81.65, 81.66 |
| Femoral fracture | | 821.00, 821.10, 821.23, 821.33 |

**Table S4. Cox regression analysis on mortality including patients dying in the first two years, 2-level definition of corticosteroid treatment, including CCI (non-rheumatologic comorbidities vs rheumatologic comorbidities only).**

|  | HR (95% CI) | Adj HR (95% IC) |
| --- | --- | --- |
| No GC | Ref | Ref |
| GC | 1 (0.59,1.70) | 1.39 (0.78,2.48) |
| Female gender |  | 0.71 (0.40,1.25) |
| Age | ***-*** | ***1.11 (1.07,1.15)*** |
| HAQ | - | 1.45 (0.98,2.14) |
| CCI >1 | ***-*** | ***1.94 (1.06,3.55)*** |
| MTX | - | ***0.49 (0.24,0.98)*** |
| ACPA | - | 1.42 (0.66,3.04) |

GC: glucocorticoids; HR: hazard ratio; CI: confidence interval; HAQ: health assessment questionnaire; CCI: Charlson Comorbidity Index; MTX: methotrexate; ACPA: anti cyclic citrullinated peptide antibodies.

**Table S5. Cox regression analysis on mortality, GC included as mean daily dose throughout the follow-up, including CCI (non-rheumatologic comorbidities vs rheumatologic comorbidities only).**

|  | HR (95% CI) | Adj HR (95% IC) |
| --- | --- | --- |
| GC | 1.10 (0.96,1.26) | 1.06 (0.90,1.24) |
| Female gender |  | 0.64 (0.35,1.16) |
| Age | ***-*** | ***1.11 (1.07,1.15)*** |
| HAQ | - | 1.32 (0.87,2.02) |
| CCI >1 | ***-*** | ***2.22 (1.20,4.11)*** |
| MTX | - | ***0.44 (0.22,0.91)*** |
| ACPA | - | 1.45 (0.65,3.25) |

GC: glucocorticoids; HR: hazard ratio; CI: confidence interval; HAQ: health assessment questionnaire; CCI: Charlson Comorbidity Index; MTX: methotrexate; ACPA: anti cyclic citrullinated peptide antibodies.

**Table S6. Cox regression analysis on GC-related hospitalization including patients dying in the first two years, 2-level definition of corticosteroid treatment, including CCI (non-rheumatologic comorbidities vs rheumatologic comorbidities only).**

|  | HR (95% CI) | Adj HR (95% IC) |
| --- | --- | --- |
| No GC | Ref | Ref |
| GC | 0.93 (0.64,1.34) | 1.07 (0.72,1.59) |
| Female gender | - | ***0.41 (0.27,0.61)*** |
| Age | ***-*** | ***1.07 (1.05,1.09)*** |
| HAQ | - | ***1.33 (1.01,1.74)*** |
| CCI >1 | ***-*** | ***1.70 (1.11,2.61)*** |
| MTX | - | 1.09 (0.65,1.84) |
| ACPA | - | 1.54 (0.93,2.54) |

GC: glucocorticoids; HR: hazard ratio; CI: confidence interval; HAQ: health assessment questionnaire; CCI: Charlson Comorbidity Index; MTX: methotrexate; ACPA: anti cyclic citrullinated peptide antibodies.
